# Unexpected findings before hospital discharge in a patient implanted with a dual‐chamber pacemaker

**DOI:** 10.1002/joa3.12399

**Published:** 2020-07-12

**Authors:** Miguel A. Arias, Paula Sánchez‐Aguilera, Marta Pachón

**Affiliations:** ^1^ Arrhythmia Unit Department of Cardiology Complejo Hospitalario Universitario de Toledo Toledo Spain

**Keywords:** bradycardia, minimizing ventricular pacing, permanent pacemaker, right bundle branch block

## Abstract

A case of a patient implanted with a dual‐chamber pacemaker in which routine ECG before discharge shown unexpected findings.

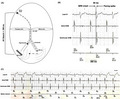

## 1 CASE PRESENTATION

**FIGURE 1:**
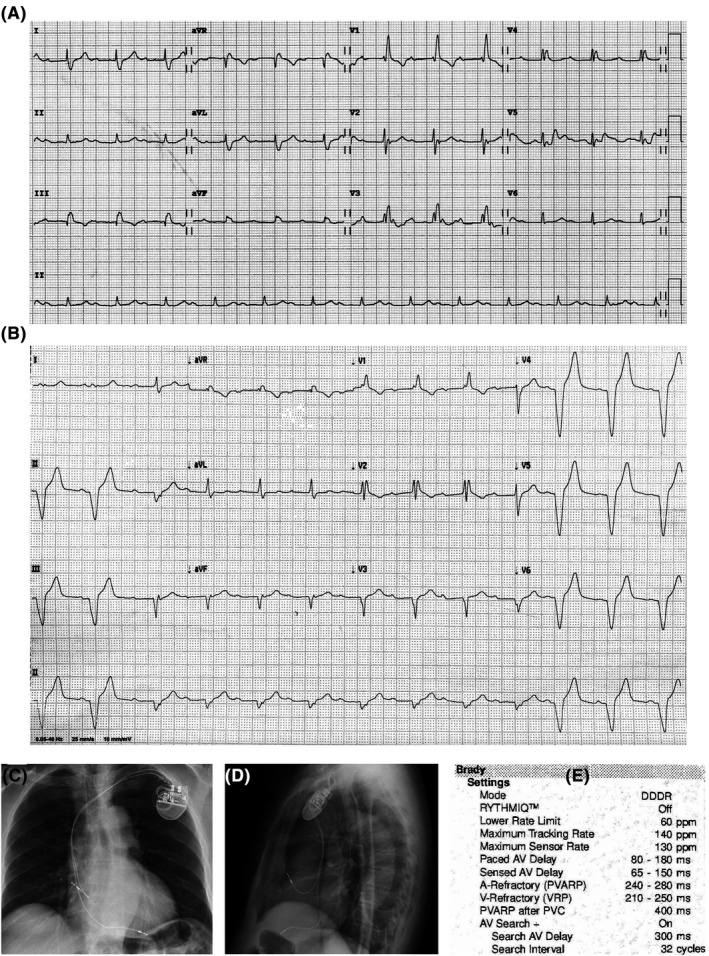
A, Baseline 12‐lead electrocardiogram and lead II rhythm strip. B, Twelve‐lead electrocardiogram and lead II rhythm strip performed the next day after pacemaker implantation. C and D, Postero‐anterior and lateral chest X‐ray projection before hospital discharge. E, Programmed pacemaker settings

A 70‐year‐old man was admitted to the hospital because of syncope caused by transient complete atrioventricular (AV) block. Any potential reversible cause was ruled out and the patient was schedule for permanent pacemaker implantation. An echocardiogram revealed the presence of preserved left ventricular systolic function. The baseline ECG is shown in Figure 1A. The patient underwent a dual‐chamber pacemaker implantation (Accolade MRI L331, Boston Scientific). Two leads were transvenously positioned at the right atrial appendage and the right ventricular (RV) apex without any complication and excellent sensing and pacing parameters. Twelve‐lead ECG was performed (Figure 1B) the next day before discharge, showed unexpected findings: The ECG showed transient presence of two different QRS morphologies.

**Question:** Is the pacemaker working properly?

## 2 DISCUSSION

Due to the very small pacemaker pulse amplitudes generated by bipolar pacemakers it is not always possible to detect pacing spikes in the ECG. Baseline ECG (Figure 1A) shows sinus rhythm at a rate of 76 bpm, PR interval of 400 ms, and RBBB with normal axis. The ECG in Figure 1B shows sinus rhythm with no visible pacing spikes. The first two beats show a PR interval of 130 ms and wide QRS‐complexes with superior axis; then a lengthening of the PR interval is shown for the next eight beats showing a narrower QRS‐complexes with RBBB, superior QRS axis and predominantly negative QRS‐complexes from V4 trough V6. For the last three beats, the shorter PR intervals with wider QRS‐complexes resumed. Therefore, the first two beats and the last three beats in Figure 1B showing a PR interval of 130 ms and wider QRS complexes with superior axis are due to atrioventricular (AV) sequential pacing or P‐synchronous ventricular pacing (indistinguishable only based on the trace by the lack of visualization of the pacing spikes). Unlike what happens in the basal ECG (Figure 1A) where RBBB also exists, in these eight beats with narrower QRS complex the QRS axis is superior with predominantly negative QRS complex in lead V6, which rules out that they are pure native QRS complexes. Despite the lack of visualization of pacing spikes, the change in QRS morphology indicates that these are fusion beats being intermediate between a native QRS and a fully paced QRS complex. Fusion beats in this case represents attempted avoidance of continuous ventricular pacing and is consistent with normal pacemaker function.

**FIGURE 2:**
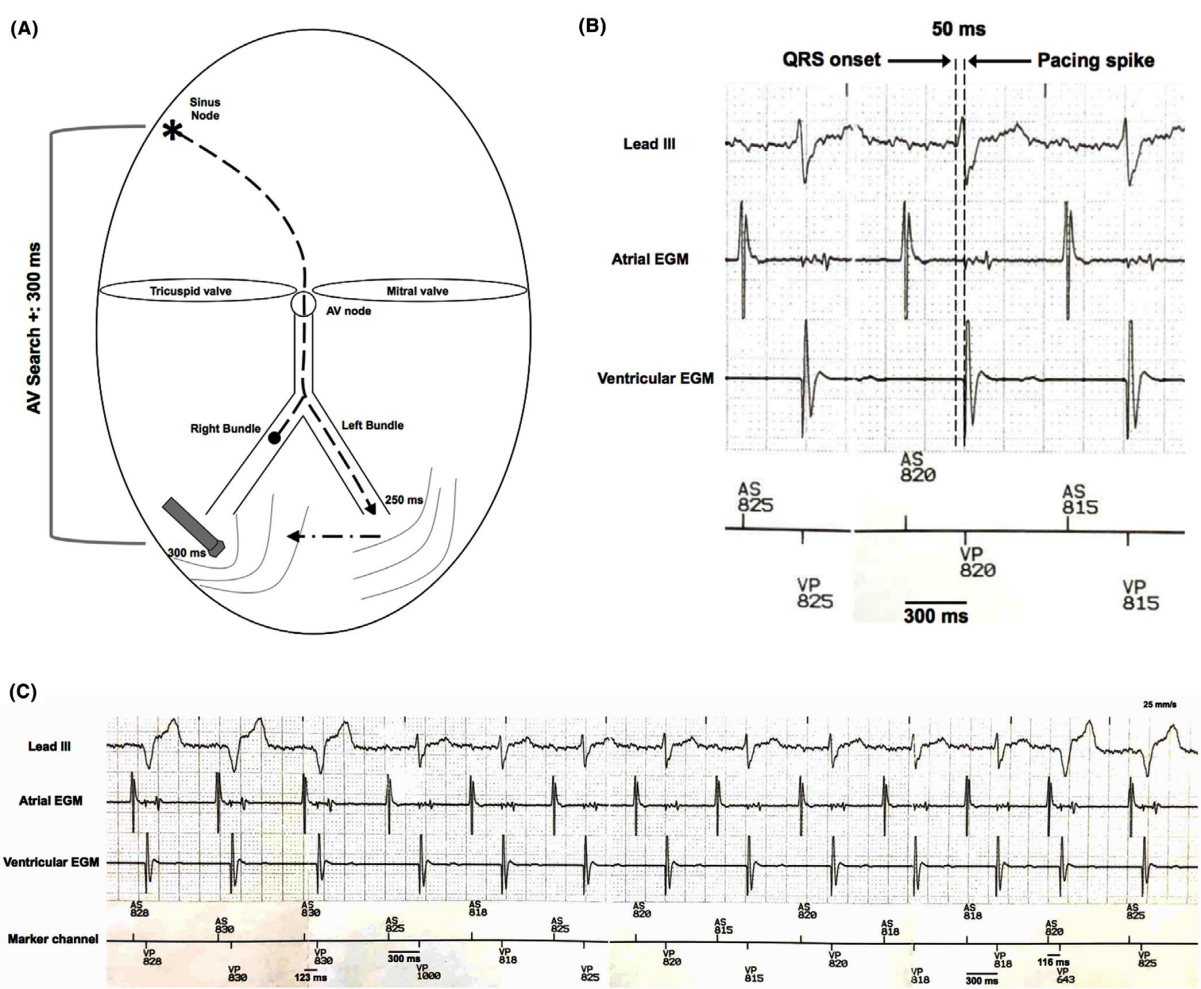
Schematic representation (A) of a fusion beat during an episode of AV delay lengthening determined by the AV Search + algorithm with its corresponding intracardiac electrograms during device interrogation (B). In (C) interrogation of the device during an episode of AV delay lengthening to check for intrinsic conduction by the AV Search + algorithm. In panel A, the sinus beat is conducted to the ventricle through the left bundle‐branch, reaching it at 250 ms; then, the activation wavefront spreads through the right ventricle where the ventricular lead is placed, although the programmed search AV delay (300 ms) of the AV Search + algorithm allows ventricular pacing to occur just before the ventricular activation wavefront is detected by the ventricular lead, producing a fusion beat. In panel B, a 50‐msec delay is observed between the onset of the surface QRS complex and sensing of the local electrical wavefront as it reaches the right ventricular lead. This delay is due to the basal right bundle branch block and allows pacing with fusion beats. The basal native atrioventricular conduction was 250 ms and the programmed AV delay during AV Search + was 300 ms In panel C, electrocardiographic lead III, atrial electrogram, and ventricular electrogram are shown. When AV Search + is turned on, the AV delay is lengthened periodically for up to 8 consecutive paced or sensed cardiac events. It remains active as long as the intrinsic PR intervals are shorter than the programmed search AV delay value (300 ms in that case). The pacemaker switches back to the programmed AV delay when the 8‐cycle search finishes without sensing intrinsic ventricular activity, resulting in fully paced QRS beats for the last three QRS complexes of the tracing. A significantly longer programmed search AV delay in the algorithm would have resulted in inhibition of pacing by sensing ventricular events, and much shorter programmed search AV delay would have resulted in fully ventricular paced beats. AV: atrioventricular; EGM: electrogram. AS: atrial sensed event; VP; ventricular paced event

Fusion beats in a patient implanted with a pacemaker are caused by the position of the sensing lead, relative to the depolarizing wavefront. In the setting of RBBB, a ventricular event will be sensed by the RV lead well beyond the onset of QRS complex on the surface ECG (Figure 2). As in our patient, there is a delay in the arrival of the activation wavefront of the spontaneous beats at the site of the ventricular lead. The pacemaker may discharge its pacing stimulus at its preset AV interval in the initial part of the QRS complex because it has not yet sensed the activation wavefront coming from the left ventricle. This does not represent undersensing; rather, it is normal sensing occurring late in the right ventricle owing to a conduction delay. The reason for the observed transient prolongation of AV interval that led to fusion beats was a programmed feature of the pacemaker called AV Search+, an algorithm created to minimize ventricular pacing, because of the well‐established deleterious effects of frequent RV pacing on clinical outcomes and device longevity.^1^ The algorithm is aimed at maximizing intrinsic AV conduction, while reducing the risks of a very prolonged PR interval (shortening and impairment of left ventricular filling, presystolic mitral regurgitation, or increased left atrial pressure^2^) by allowing AV conduction to occur beyond the programmed AV delay. In some patients, symptoms similar to pacemaker syndrome are provoked by the adverse prolongation of the AV electromechanical sequence. Moreover, disadvantages of long AV delay on pacemaker time‐cycle include lower 2:1 block point, possible occurrence of repetitive non–reentrant ventriculoatrial synchrony (RNRVAS) or pacemaker mediated endless loop tachycardia. The AV delay is lengthened periodically to a programmed maximum limit (300 ms in our patient) for up to eight consecutive paced or sensed cardiac cycles. If intrinsic conduction is identified, the AV delay is automatically reprogrammed to allow for intrinsic AV conduction, otherwise the algorithm switches back to DDD if intact AV conduction is not detected, as in the preset case. Hence, the pacemaker was working properly in the present case.

The case highlights the importance of clinicians knowing basic concepts of modern algorithms in cardiac implantable electronic devices^3^ and pacing electrocardiography to avoid misinterpretations that can determine unnecessary or wrong actions.

## CONFLICT OF INTEREST

The authors declare no conflict of interests for this article.
